# Soil biochemical responses to nitrogen addition in a secondary evergreen broad-leaved forest ecosystem

**DOI:** 10.1038/s41598-017-03044-w

**Published:** 2017-06-05

**Authors:** Yong Peng, Guangsheng Chen, Guantao Chen, Shun Li, Tianchi Peng, Xirong Qiu, Jie Luo, Shanshan Yang, Tingxing Hu, Hongling Hu, Zhenfeng Xu, Li Liu, Yi Tang, Lihua Tu

**Affiliations:** 1 College of Forestry, Sichuan Agricultural University, 211 Huimin Road, Chengdu, 611130 China; 2 Ecological Security and Protection Key Laboratory of Sichuan Province, Mianyang Normal University, 166 Mianxing West Road, Mianyang, 621000 China; 3 Personnel Department, Sichuan Agricultural University, 211 Huimin Road, Chengdu, 611130 China; 4 College of Horticulture, Sichuan Agricultural University, 211 Huimin Road, Chengdu, 611130 China

## Abstract

In order to investigate the effects of N deposition on soil biochemistry in secondary forests, one N addition experiment was conducted in a secondary evergreen broad-leaved forest in the western edge of Sichuan Basin, with the highest level of background N deposition (about 95 kg N ha^−1^ yr^−1^) in China. Three N treatment levels (+0, +50, +150 kg N ha^−1^ yr^−1^) were monthly added to soil surface in this forest beginning in April 2013. Soil biochemistry and root biomass of the 0–10 cm soil horizon were measured from May 2014 to April 2015. Soil respiration was measured for two years (September 2013 to August 2015). It was showed that N additions were correlated to significantly lower soil pH, microbial biomass C (MBC) concentration, MBC/microbial biomass N (MBN) ratio, root biomass, and soil respiration rate, and significantly higher concentrations of ammonium (NH_4_^+^) and nitrate (NO_3_^−^). These results indicate that N additions had a significant effect on the size of soil microbial community. In addition, soil C storage may potentially increase due to the dropped soil C release under N addition.

## Introduction

Over the last century, anthropogenic annual nitrogen (N) emissions, derived from human activities such as combustion of fossil fuels and biofuels, and the production and use of nitrogen fertilizer, have come to exceed that produced by all natural terrestrial systems. This has resulted in a rapid increase in atmospheric N deposition^1, 2^. Worldwide, over 11% of natural vegetation presently receives N deposition in excess of the critical load threshold (10 kg N hm^−2^ yr^−1^)^3^. It has been reported that increased N deposition has substantial impacts on terrestrial ecosystems, including soil carbon (C) and N cycling, microflora health, and ecosystem functions and services^4–7^. Generally, the net primary productivity (NPP) of most terrestrial ecosystems is often limited by N; thus, N additions tend to increase NPP in these ecosystems^8^. A number of studies have reported that N additions significantly increase ecosystem NPP^9–11^, and aboveground biomass^12, 13^. This would potentially increase soil C storage^14, 15^. However, the responses of belowground biomass to N addition were inconsistent across different studies because of the inherent variability in forest properties and environmental factors. Positive^10, 16^, neutral^12, 17^ and negative^18, 19^ effects of added N on belowground biomass were reported by previous studies. Thus, the responses of belowground C to N additions remain unclear.

Soil C storage in forest ecosystems is dependent on the balance of C input through plant litter and C emissions through organic matter decomposition. The response of C input to N addition is generally positive^20, 21^. Many studies have indicated that N deposition can increase^10, 22^ or decrease^23, 24^ soil C emissions by affecting microbial activity, soil biochemistry, and plant root characteristics^25–27^. The response of the C cycle to N addition is primarily linked to changes in the soil environment caused by N deposition. Previous studies indicated that increased N deposition generally results in soil acidification^28, 29^, and changes soil enzyme activity, microbial activity and nutrient cycling^26, 30, 31^. All of these effects are likely to affect soil C dynamics.

Currently, forest N deposition research is primarily concentrated in temperate and boreal forests. Relatively few such studies have been conducted in tropical and subtropical forests, particularly secondary forests. Secondary forests are plant communities that naturally regenerate after complete anthropogenic forest clearance. In China, due to over-exploitation and excessive harvest in the past, primary forests have gradually disappeared and have been replaced by large areas of secondary forests^32^. These represent an important component of forest resources in China^33^. Many studies indicated that restored secondary forests played an important role in terrestrial ecosystems’ net C sinks over the past few decades^34–36^. It was reported that, on a global scale, secondary forests contributed an estimated 0.35–0.6 Gt C yr^−1^ to terrestrial C sinks in the 1990s^35^, while N deposition contributes about 0.13 Gt C yr^−1^ to the total secondary forest C sinks^36^. Although the stimulatory effect of N deposition on the growth of secondary forests is the main explanation for the increase in forest C sequestration in recent years^36^, many studies have indicated that the effects of N deposition on various forests are not consistent, and eventually, some forests may transition from N-limited to N-saturated ecosystems^37^.

The large ecotone in southwestern China, on the western edge of the Sichuan Basin has a cloudy, wet climate. The average wet N deposition in this region from 2008 to 2010 was about 95 kg N ha^−1^ yr^−1^, which is considerably higher than the corresponding mean estimates for other areas in China, and in the United States and Western Europe^38–40^. Several possible reasons can be used to explain the high level of wet N deposition rate in this region. Firstly, the Sichuan Basin is an important industrial–agricultural economic region in southwest China. The reactive N creation in this region significantly increased because of the rapid development in economy. And reactive N released from the Sichuan Basin could be transported to the western edge of the Sichuan Basin by monsoon. Secondly, due to the rise in elevation, warm moist air from the Sichuan Basin is readily condensed into rain on the western edge of the Sichuan Basin. Therefore, there is abundant rainfall along the western edge of the Sichuan Basin (known as the “rainy zone of west China”). Thus, reactive N with rainfall may be deposited substantially in this special zone. It has been predicted that the largest increases in N deposition in the world will occur in this region over the next few decades^3^.

It is well believed that excessive N deposition induces a considerable burden on the functions and structures of ecosystems. Previously, we conducted simulated N deposition tests in *Pleioblastus amarus* and hybrid bamboo (*Bambusa pervariabilis* × *Dendrocalamopsis daii*) plantations and several other forest plantations in this region. The results from these studies indicated that N additions increased the rates of soil N mineralization and nitrification, the concentrations of NH_4_^+^ and NO_3_^−^, microbial biomass carbon, fine root biomass, and mean annual soil respiration rate. On the other hand, litter decomposition rates and soil pH decreased, with mixed effects on soil enzyme activities^10, 31, 41–43^. These results indicate that such a high level of N deposition may have severely affected many ecosystem properties and processes in this area. Notably, a large area of secondary forest was naturally generated from a sapling surviving from 1956 when the virgin forest in this region was destroyed. There are significant differences in stand age, soil development, soil fertility and ecosystem structure among primary forests, secondary forests and forest plantations. Thus, the responses of the three types of forests to N addition were potentially different. Secondary forest is the most important forest type in high N deposition region in China. What are the ecological consequences of continuous increasing N deposition for local secondary forest, in this region with high level of background N deposition is still not very clear. Therefore, an experimental N addition study was conducted in a subtropical secondary evergreen broad-leaved forest on the western edge of the Sichuan Basin, China. The aim of this study was to understand the effects of elevated N deposition on soil C status, nutrient availability, microbial properties and soil enzyme activities in a secondary forest ecosystem receiving high level of ambient N deposition.

## Materials and Methods

### Site description

The N addition experiment was conducted in a secondary evergreen broad-leaved forest in Wawushan Mountain National Forest Park, situated in Hongya, Sichuan Province, China (29°32′35″N, 103°15′41″E, altitude 1 600 m). The area experiences a monsoon-influenced, subtropical highland climate. The annual mean temperature is 10 °C, with lowest monthly value (−0.9 °C) in January and highest value (22.5 °C) in July. The annual rainfall and evapotranspiration are respectively 2 323 mm and 467 mm, and the annual average relative humidity is 85% to 90%. The soil is classified as a Lithic Dystrudepts (according to USDA Soil Taxonomy), and the nature of bedrock is granite. The average soil depth to bedrock is deeper than 1 m. Before the primary forest was destroyed in 1956, the site was representative of the mid-subtropical evergreen broad-leaved biome characterizing the study area, consisting of *Castanopsis platyacantha* and *Schima sinensis*. No further disturbance occurred after 1956, allowing the survivors to naturally recover into mature secondary evergreen broad-leaved forest. At the study site, the average plant density was 725 stems per hectare and the mean diameter at the breast height (DBH) was 23.5 cm. This forest is dominated by the tree species *C. platyacantha* and *S. sinensis*, DBH of them were 23.8 cm and 25.4 cm, respectively, and *C. platyacantha* is the most important constrictive species with the highest importance value of 56.91; the shrub species *Ilex purpurea* and *Eurya japonica*, and the sparsely distributed herb species *Cyperus rotundus*.

### Soil and litter chemistry before N treatment

In November 2012, thirty six litter samples were randomly taken from surface using a metal frame (50 cm × 50 cm) study site. At the same time, thirty six soil profiles (1 m) were dug. Each soil profile was divided into four layer (0–10 cm, 10–40 cm, 40–70 cm, 70–100 cm), since the thickness of the top organic soil is about 10 cm. Soil samples in each layer were collected using a small shovel for chemical analysis. For measuring soil bulk density (g cm^−3^), an undisturbed soil was collected using a 100 cm^3^ cutting ring. Litter samples were dried to constant weight at 65 °C and weighed. Then each litter sample was ground using a Wiley mill with a 1-mm mesh screen. Soil samples were air-dried, ground and sieved through a 2 mm mesh for determining soil potential acidity, and sieved through a 0.25 mm mesh for measuring soil total organic carbon (TOC), total nitrogen (TN), total phosphorus (TP) and total potassium (TK). Total organic carbon of litter and soil was determined by the dichromate digestion method, and TN was measured by the Kjeldahl method. For determining TP and TK concentrations, litter and soil samples were digested by perchloric acid-sulfuric acid (HClO_4_-HSO_4_) and sodium hydroxide (NaOH), respectively. Then TP and TK were determined using colorimetry an atomic absorption spectrophotometer (TAS-986, PGENERAL, Beijing, China), respectively. Soil potential acidity (pH-KCl) was determined by a glass electrode in 1*M* potassium chloride (KCl) extracts. The results were shown in Table 1.

**Table 1:** Physicochemical properties of soil and leaf litter in a secondary evergreen broad-leaved forest near Wawushan Mountain, SW China (Mean ± 1 SE, *n* = 36).

Soil depth (cm)	Soil potential acidity (pH-KCl)	Soil bulk density (g cm^−3^)	C (g kg^−1^)	Total N (g kg^−1^)	Total P (g kg^−1^)	Total K (g kg^−1^)	C/N	N/P
Leaf litter	—	—	443.0 ± 4.2	9.9 ± 0.2	1.0 ± 0.1	1.0 ± 0.1	45.3 ± 1.0	15.5 ± 1.2
0–10	3.19 ± 0.02	0.4 ± 0.0	121.9 ± 6.7	5.9 ± 0.3	0.5 ± 0.0	13.4 ± 0.6	20.6 ± 0.6	12.2 ± 0.5
10–40	3.76 ± 0.03	0.7 ± 0.0	26.6 ± 1.5	1.6 ± 0.1	0.3 ± 0.0	16.9 ± 0.5	16.9 ± 0.5	6.7 ± 0.3
40–70	3.97 ± 0.01	0.9 ± 0.0	12.6 ± 0.7	0.9 ± 0.1	0.2 ± 0.0	18.7 ± 0.7	14.3 ± 0.6	5.1 ± 0.3
70–100	4.03 ± 0.01	1.0 ± 0.0	7.8 ± 0.5	0.7 ± 0.0	0.2 ± 0.0	18.7 ± 0.8	12.4 ± 0.6	3.4 ± 0.2

### Experimental design

Nine 20 m × 20 m plots were established within the study site in October 2012, at intervals of more than 20 m. Plots were divided into three treatments with three plots assigned to each: low nitrogen treatment (LN, +50 kg N ha^−1^ yr^−1^), high nitrogen treatment (HN, +150 kg N ha^−1^ yr^−1^), and ambient nitrogen/control (CK, +0 kg N ha^−1^ yr^−1^). Consequently, the cumulated doses received by CK, LN and HN plots was 95, 145, and 245 kg N ha^−1^ yr^−1^, respectively. The LN and HN treatments simulate scenarios of nitrogen deposition increased by 50% and 150%. All nitrogen treatments plots were randomly selected. Beginning in April 2013, ammonium nitrate (NH_4_NO_3_) solution was applied to the soil surface monthly and continued for the duration of the study (April 2013 to August 2015). In each month, the fertilizer was weighed, dissolved in 10 L of water, and applied to each plot using a sprayer. The control plots received an equivalent volume of water without fertilizer.

### Litter fall and fine root biomass

Litter was collected monthly from ten 1 m × 1 m nylon mesh nets installed at randomly selected positions in each plot from May 2014 to April 2015. Because of winter snow cover, litter from January, February and March were collected as one composite sample at the end of March. Each litter was dried to constant weight at 65 °C and weighed.

The soil core method was used to determine root biomass. Root samples were taken in May, June, August, October and November of 2014 and April 2015. Three healthy mean trees (*C. platyacantha*) with DBH of about 23.8 cm were selected as target trees in each plot. For each sample, two soil cores at the top 10 cm were collected at 1 m from each designated sample tree using a soil auger 5 cm in diameter. Three trees were collected in each plot. Samples were placed into plastic bags and store at −4 °C. Roots were separated from soils by washing and sieving with a 0.25 mm sieve. Live roots were distinguished from dead roots by color and flexibility. Roots of *C. platyacantha*, shrubs and grasses were distinguished from each other by color and morphology. For determining root biomass, roots were dried at 65 °C for 48 h and weighed. Root biomass was expressed as the weight of roots per unit volume of soil (g m^−3^).

### Soil biochemical characteristics measurements

Nine composite samples were obtained from the experimental site in May, September and November 2014, and April 2015. Each composite sample comprised five subsamples of the organic soil layer (about 0–10 cm) randomly collected from each plot with a soil auger. After removing the visible roots using tweezers, the soil samples were ground, sieved through a 2 mm mesh, and stored at 4 °C for analysis within 1 week. For measuring soil total organic carbon (TOC) and total nitrogen (TN), air-dried subsamples were ground and passed through a 0.25 mm sieve.

Total organic carbon was determined by the dichromate digestion method, while TN was measured using the Kjeldahl method. Soil ammonium nitrogen (NH_4_^+^) and nitrate nitrogen (NO_3_^−^) were extracted with a 2 M KCl solution and measured with colorimetry. Soil microbial biomass carbon (MBC) and soil microbial biomass nitrogen (MBN) were measured using the 24-h chloroform fumigation extraction technique using a total C/N analyzer (Shimadzu model TOC-VcPH +TNM-1, Kyoto, Japan). The MBC was calculated as the difference in extractable C between fumigated and unfumigated soils, divided by 0.45. The MBN also was calculated as the difference in extractable C between fumigated and unfumigated soils, but divided by 0.54. Soil available phosphorous (AP, include water soluble P and inorganic P) was extracted with mixed solution of 0.05 M HCl and 0.0125 M H_2_SO_4_ and measured with colorimetry. Soil available potassium (AK) was extracted with 1 M ammonium acetate (CH_3_COONH_4_) and measured using an atomic absorption spectrophotometer (TAS-986, PGENERAL, Beijing, China). Here, in order to comparing with other studies, soil pH was determined by a glass electrode in aqueous extracts (pH-H_2_O).

Urease activity was measured spectrophotometrically according to Sinsabaugh *et al*.^44^. Invertase activity was measured using the method described by Frankeberger Jr and Johanson^45^. Protease activity was determined by the method of Zhang^46^ using sodium caseinate as substrate. The activity of acid phosphatase (AcPh) was measured following a publication of Saiya-Cork *et al*.^47^ using 4-methyumbelliferyl (MUB) phosphate as substrate. The activity of nitrate reductase (NR) was measured spectrophotometrically according to Zhang^46^ with little modification. Enzyme activity was calculated as the μmoles of substrate converted per hour per gram of dried soil.

### Soil respiration measurements

In July 2013, five polyvinyl chloride (PVC) loops (20 cm inside diameter, 8 cm height) were randomly installed in each plot. The loops were inserted into the soil to a 6 cm depth. Soil respiration was measured monthly using a Li-8100 soil CO_2_ flux system (LI-COR Inc., Lincoln, NE, USA) from September 2013 to August 2015. All measurements were recorded between 12:00 and 15:00 (local time) on each measurement date.

### Statistical analyses

All statistical analyses were performed using SPSS 20.0 for Windows (SPSS Inc., Chicago, USA). Repeated measures ANOVA with Fisher’s LSD tests were used to examine litterfall, root biomass, TOC, TN, NH_4_^+^, NO_3_^−^, MBC, MBN and soil respiration. Relationships between soil properties were determined by using Pearson correlation coefficients. We determined the relationship of soil respiration rate to root biomass or soil MBC content using linear regression. Significant effects were determined at *α* = 0.05.

## Results

### Litterfall and root biomass

**Figure 1:**
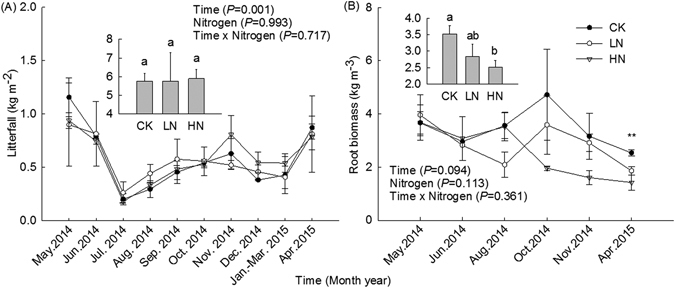
Litterfall mass and root biomass under different nitrogen treatments in a secondary evergreen broad-leaved forest ecosystem in southwestern China, from May 2014 to April 2015. Histograms indicate annual mean values. Plots received three levels of N addition, while control plots received none. Monthly applications of NH_4_NO_3_ began in April 2013. Values are means ± SE. Bars indicate ± 1 SE, *N* = 3. The results of repeated measures ANOVAs are shown for each parameter. Double asterisks (**) indicate a significant difference between the control and at least one experimental N treatment at *P* < 0.01. Different letters denote significant differences (one-way ANOVA with Fisher’s LSD test, *P* < 0.05) between treatments.

Litterfall mass at the experimental site displayed evident seasonal variation and peaked in May (Fig. 1A), while root biomass of *Castanopsis platyacantha* was relatively stable (Fig. 1B). The average sum of litterfall over the period May 2014 to April 2015 was 5.8 ± 0.4 kg m^−2^ yr^−1^. Repeated measures ANOVA revealed that simulated N additions had no significant effect on litterfall. The average root biomass at control plots was 3.5 ± 0.2 kg m^−3^, but was 19% and 29% lower under LN and HN treatments, respectively. The latter difference was significant (*P* = 0.048).

### Soil nutrient availability

**Figure 2:**
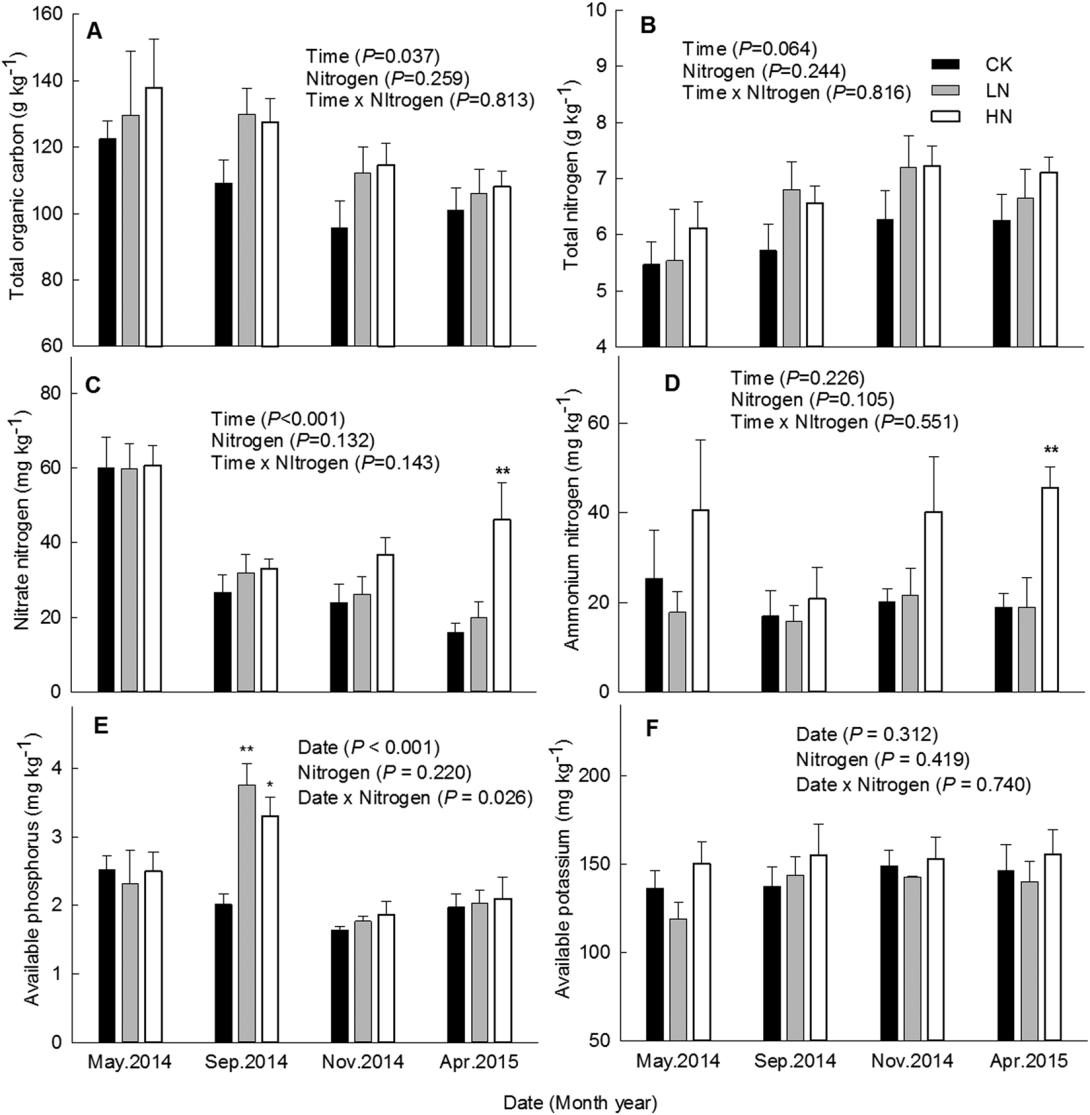
Soil nutrient availability under different nitrogen treatments in a secondary evergreen broad-leaved forest ecosystem, southwestern China. Values are means ± 1 SE. Error bars indicate ± 1 SE, *N* = 3. The results of repeated measures ANOVAs are shown for each parameter. Asterisk (*) indicate a significant difference between the control and experimental N treatment at *P* < 0.05; double asterisks (**) indicate a significant difference between the control and experimental N treatment at *P* < 0.01.

Repeated measures ANOVA indicated that there were significant seasonal patterns in soil TOC, NO_3_^−^ and AP concentrations (*P* < 0.05, Fig. 2A,C,E); the addition of N significantly changed the seasonal pattern of AP (*P* = 0.026, Fig. 2E). Meanwhile, seasonal variations in the concentrations of TN, NH_4_^+^ and AK were not significant (Fig. 2B,D,F). In general, there were no significant differences between N treatments and the control with respect to the concentrations of soil TOC, TN, AP and AK (Table 2). In the HN treatment, soil NO_3_^−^ and NH_4_^+^ concentrations were significantly higher (39% and 80%, respectively), relative to the control. High concentrations of N significantly increased the availability of inorganic N at the organic horizon. Correlation analysis indicated that soil TOC content was significantly positively correlated with both concentrations of NO_3_^−^ and AP (Table 3).

**Table 2:** Results of repeated measures ANOVA of soil nutrient availability, microbial properties and pH (Mean ± 1 SE, *n* = 3).

Treatments	TOC (g kg^−1^)	TN (g kg^−1^)	NO_3_^−^ (mg kg^−1^)	NH_4_^+^ (mg kg^−1^)	AP (mg kg^−1^)
CK	107.1 ± 6.6a	5.9 ± 0.4a	31.7 ± 4.3a	20.4 ± 4.0a	2.0 ± 0.1a
LN	119.4 ± 6.6a	6.6 ± 0.3a	34.5 ± 1.0a	18.6 ± 3.6a	2.5 ± 0.2a
HN	122.0 ± 4.9a	6.8 ± 0.3a	44.2 ± 5.0b	36.8 ± 7.8b	2.4 ± 0.2a
	**AK (mg kg** ^**−1**^ **)**	**MBC (g kg** ^**−1**^ **)**	**MBN (g kg** ^**−1**^ **)**	**MBC/MBN**	**pH (water)**
CK	142.2 ± 8.7a	5.4 ± 1.0a	0.2 ± 0.0a	25.6 ± 4.0a	3.91 ± 0.01a
LN	136.2 ± 5.8a	2.6 ± 0.1b	0.2 ± 0.0a	12.4 ± 1.2b	3.78 ± 0.02b
HN	153.2 ± 10.6a	3.4 ± 0.3b	0.2 ± 0.0a	15.1 ± 0.8b	3.73 ± 0.03b

The soil samples were taken from the organic top layer (about 0–10 cm).

Different letters indicate significant differences among different treatments (Repeated measures ANOVA with Fisher’s LSD test, *α* = 0.05).

**Table 3:** Results of Pearson correlation analysis of soil nutrient availability, microbial properties and pH in a secondary evergreen broad-leaved forest ecosystem (*n* = 27).

	TOC	TN	NO_3_^−^	NH_4_^+^	AP	AK	MBC	MBN	MBC/MBN
TN	0.35								
NO_3_^−^	0.50**	−0.16							
NH_4_^+^	0.26	0.31	0.32						
AP	0.57**	0.09	0.27	−0.11					
AK	0.23	0.43**	−0.10	0.14	0.16				
MBC	−0.06	−0.43**	0.06	−0.16	0.11	−0.26			
MBN	0.30	0.17	0.14	0.06	0.13	−0.05	0.11		
MBC/MBN	−0.19	−0.47**	−0.01	−0.18	0.03	−0.24	**0.93** ^**^	−0.22	
pH	−0.74**	−0.01	−0.72**	−0.33	−0.52**	0.11	−0.05	−0.24	0.08

*Correlation is significant at the 0.05 level (2-tailed).

**Correlation is significant at the 0.01 level (2-tailed).

### Soil microbial properties and pH

**Figure 3:**
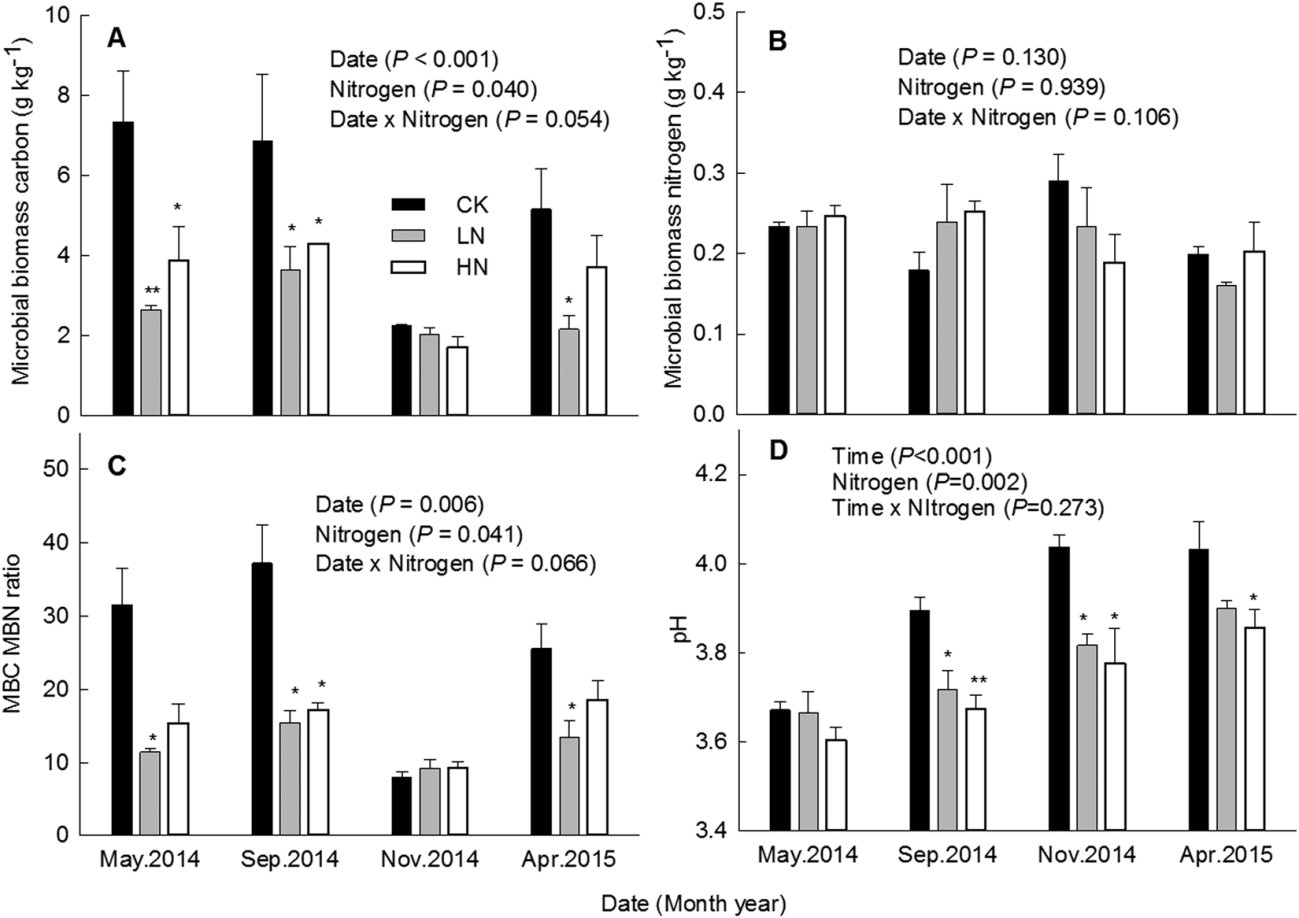
Soil microbial properties and pH values under different nitrogen treatments in a secondary evergreen broad-leaved forest ecosystem, southwestern China. Values are means ± 1 SE. Error bars indicate ± 1 SE, *N* = 3. The results of repeated measures ANOVAs are shown for each parameter. Asterisk (*) indicate a significant difference between the control and experimental N treatment at *P* < 0.05; double asterisks (**) indicate a significant difference between the control and experimental N treatment at *P* < 0.01.

The MBC concentration, ratio of MBC to MBN, and pH displayed significant temporal variation (*P* < 0.01), whereas the concentration of MBN was relatively stable (*P* = 0.130, Fig. 3). The mean concentrations of MBC in CK, LN and HN treatments were 5.40 ± 0.97, 2.62 ± 0.13 and 3.40 ± 0.33 g kg^−1^, respectively, with the differences being significant (Table 2). The effect of N addition on MBN concentration was not significant. The ratio of MBC to MBN was 51% and 41% lower under LN and HN, respectively, relative to the control (*P* < 0.05). The average pH at the organic horizon in control plots was 3.91 ± 0.01, while plots under N treatments exhibited a significantly (*P* = 0.002) lower pH. Correlation analysis showed that the concentration of MBC and the ratio of MBC to MBN were both negatively correlated with soil TN concentration, and that these correlations were highly significant. Similarly, highly significant negative correlations were observed between soil pH and the concentrations of soil TOC, NO_3_^−^ and AP (Table 3).

### Soil enzyme activities

**Figure 4:**
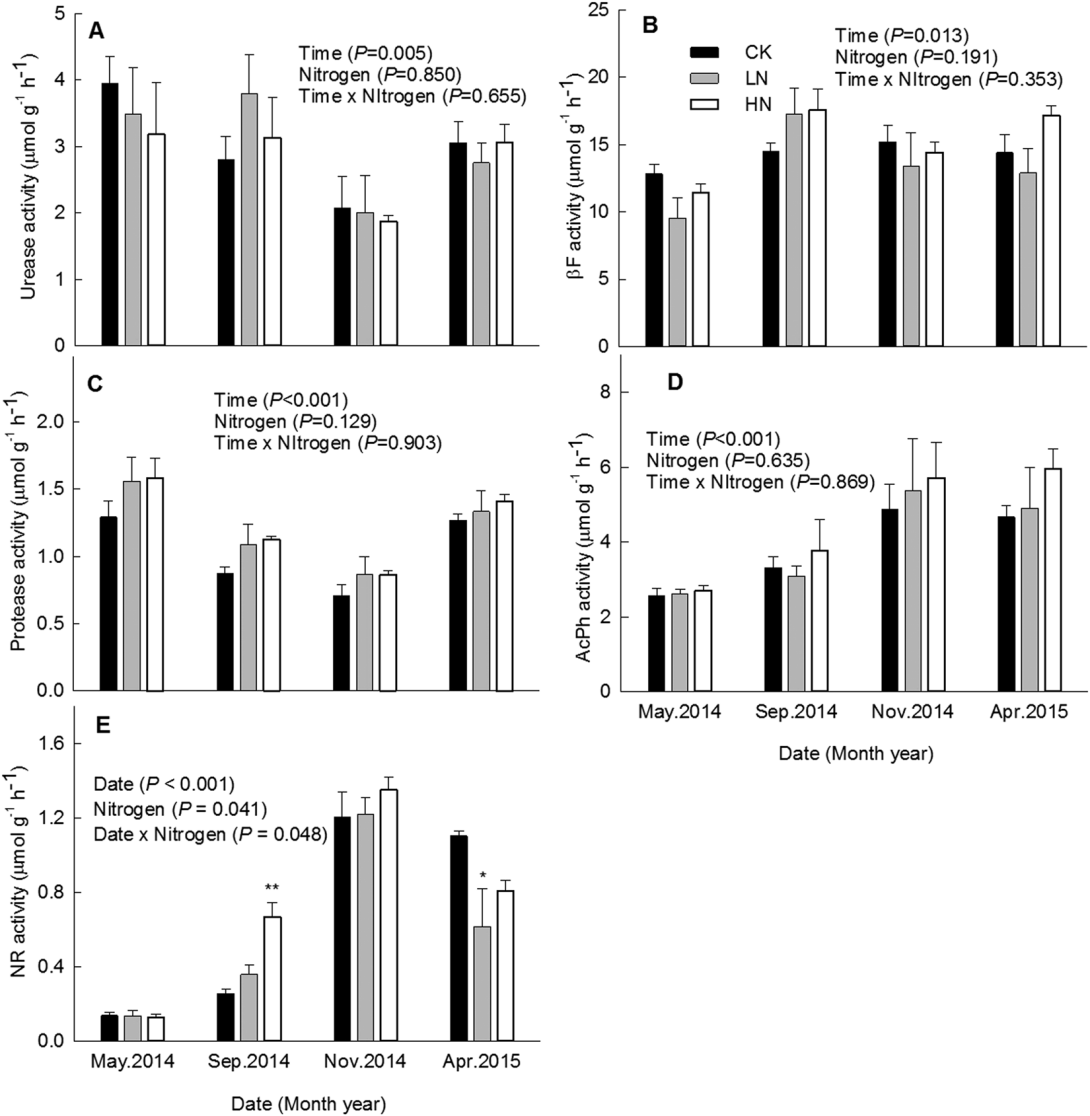
Soil enzyme activities under different nitrogen treatments in a secondary evergreen broad-leaved forest ecosystem in southwestern China. Values are means ± 1 SE. Error bars indicate ± 1 SE, *N* = 3. The results of repeated measures ANOVAs are shown for each parameter. Asterisk (*) indicate a significant difference between the control and experimental N treatment at *P* < 0.05; double asterisks (**) indicate a significant difference between the control and experimental N treatment at *P* < 0.01.

The activities of all five soil enzymes assessed displayed significant temporal variation (*P* < 0.05, Fig. 4); N addition significantly altered temporal variations in NR activity (*P* = 0.048, Fig. 4E). Repeated measures ANOVA indicated that the addition of N was associated with significantly lower activity of NR (14% lower in LN relative to the control). No significant correlations were detected with respect to activities of urease, invertase, protease and AcPh (Table 4).

**Table 4:** Results of repeated measures ANOVA of soil enzyme activities (*μ*mol g^−1^ h^−1^, Mean ± 1 SE, *n* = 3).

Treatments	Urease	Protease	NR	Invertase	AcPh
CK	3.0 ± 0.2a	1.0 ± 0.0a	0.7 ± 0.0a	14.2 ± 0.8a	3.9 ± 0.3a
LN	3.0 ± 0.4a	1.2 ± 0.1a	0.6 ± 0.0b	13.3 ± 0.7a	4.0 ± 0.7a
HN	2.8 ± 0.3a	1.2 ± 0.0a	0.7 ± 0.0a	15.1 ± 0.2a	4.5 ± 0.5a

The soil samples were taken from the organic top layer (about 0–10 cm).

NR: nitrate reductase; invertase: *β*-Fructofuranosidase; AcPh: acid phosphatase. Different letters indicate significant differences among different treatments (Repeated measures ANOVA with Fisher’s LSD test, *α* = 0.05).

### Soil respiration

**Figure 5:**
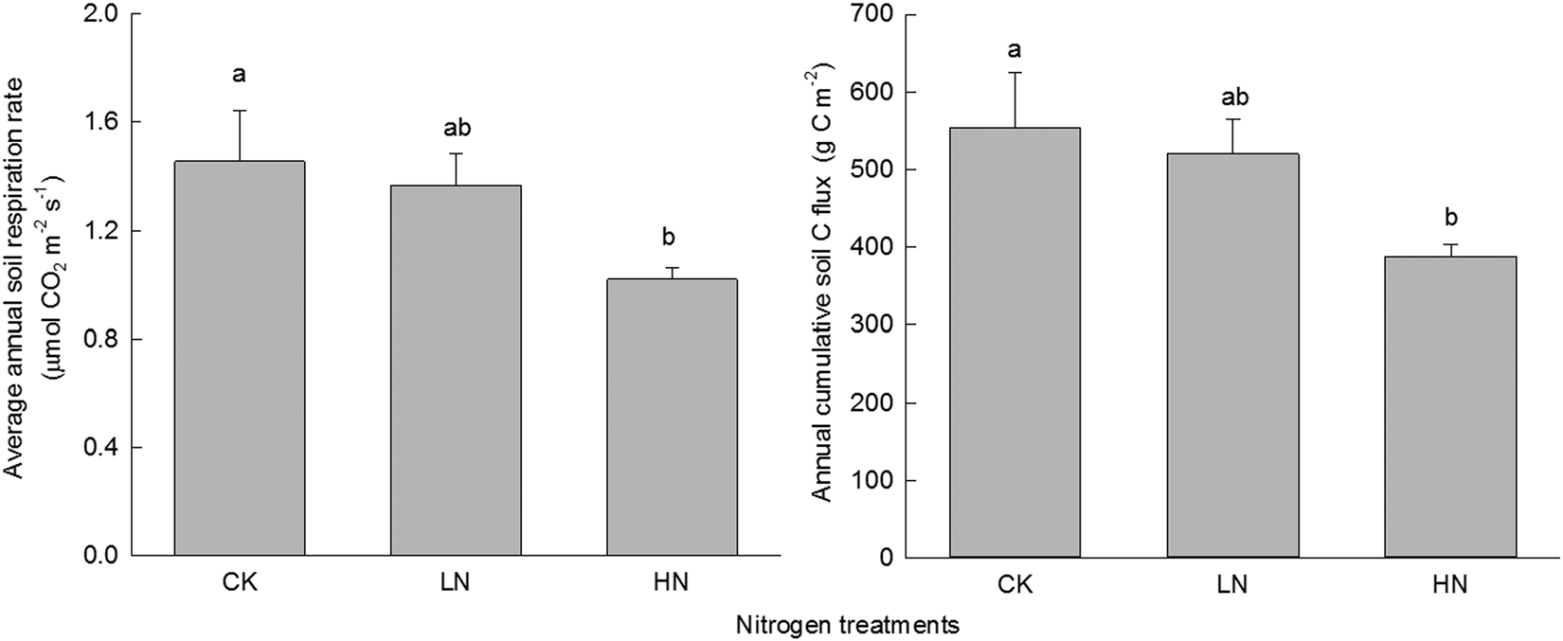
Annual mean soil respiration rate and cumulative CO_2_-C flux under different nitrogen treatments in southwestern China. Values are means ± 1 SE. Error bars indicate ± 1 SE, *N* = 3. Different letters denote significant differences (one-way ANOVA with Fisher’s LSD test, *P* < 0.05) between treatments.

**Figure 6:**
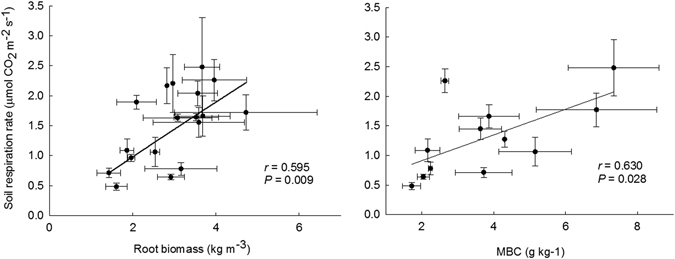
Relationship between soil respiration rate and root biomass or soil MBC content.

The mean soil respiration rate was 1.46 ± 0.19 μmol CO_2_ m^−2^ s^−1^ (63 ± 8 mg C m^−2^ h^−1^) in the control plots (Fig. 5). Compared with the control, the average respiration rates in the HN treatment were 30% lower (*P* < 0.05). From May 2014 to April 2015, the cumulative CO_2_-C flux in the CK, LN and HN treatments were 553.5 ± 71.9, 520.4 ± 45.0 and 387.9 ± 15.7 g C m^−2^ yr^−1^, respectively. Correlation analysis indicated that the soil respiration rate was significantly positively correlated with root biomass (*r* = 0.595, *P* = 0.009) and soil MBC (*r* = 0.630, *P* = 0.028, Fig. 6).

## Discussion

In the current study, N addition was associated with significantly lower soil pH, MBC concentration, MBC/MBN ratio, root biomass, and soil respiration rate. Conversely, significant positive correlations were detected with respect to the concentrations of TOC, TN, NH_4_^+^ and NO_3_^−^.

Several previous studies reported that N deposition can result in soil acidification in terrestrial ecosystems^15, 28, 29, 48^, which was confirmed by our study. Nitrogen addition was associated with heightened soil NH_4_^+^ concentration, which can be expected to accelerate nitrification^6, 43^. This is because NH_4_^+^ is the substrate of nitrification and the nitrification rate depends on soil NH_4_^+^ concentration. Moreover, heightened soil NH_4_^+^ buffered the fierce competition between nitrobacteria and plant uptake and heterotrophic microorganisms’ immobilization, which thereby promoted nitrification. During the process of nitrification, when NH_4_^+^ is transformed into NO_3_^−^, two moles of H^+^ are released into the soil per mole of NH_4_^+^ nitrified. Although ammonification process (i.e. organic matter degradation with production of NH_4_^+^) consumes one H^+^, which to some extent buffers acidification, increased soil NH_4_^+^ enhanced the substrate of nitrification that acidified soils. In addition, redundant soil NO_3_^−^ could leach out of soils, leading to the loss of metal cations based on the charge balance in soil solutions, weakening their buffering against soil acidification^29^. Besides, increased NH_4_^+^ concentration may promote plant N uptake, in which a mole of H^+^ is released per mole of NH_4_^+^ assimilated by plant roots. Therefore, N additions result in the accumulation of H^+^ in the soil and make the metal cations easy to leach out of soils, leading to acidification. A highly significant negative correlation between soil pH and NO_3_^−^ concentration (*r* = −0.720, *P* < 0.01) was observed, which suggests that a lower soil pH is associated with accelerated nitrification. However, other studies found that N additions had no significant influence on soil pH, and conjectured that enhanced production of certain metabolites by soil microorganisms could have had a neutralizing or buffering effect^30^.

Our data indicated that high levels of N addition considerably reduced fine root biomass of *C. platyacantha*. Based on the cost-benefit analysis that more resources will be allocated to aboveground biomass when soil resource availability is enriched, proportionately less C will be present in belowground biomass; this results in a decrease in root biomass under N additions^49, 50^. Other potential causes of a decline in root biomass are soil acidification and aluminium (Al) toxicity^51^. The H^+^ released in soil can rapidly react with Al in the soil mineral lattice, which will lead to a sharp increase in Al^3+^ in the soil solution^52^. Several studies have shown that Al^3+^ and H^+^ both have toxic effects on root growth and antagonistic effects on ion uptake^51^. Unsurprisingly, root biomass was significantly lower in N-treated plots.

Our results signified that N deposition significantly decreased soil MBC, which is consistent with the conclusion that N enrichment decreases soil microbial biomass in many ecosystems^26, 53^. Several potential mechanisms may help to explain this. Firstly, N additions reduced root biomass, metabolism and C exudate production, with consequent effects on rhizosphere microorganism activity and biomass. Secondly, high N additions may lead to a condensation of organic compounds with N-containing compounds and/or accumulation of compounds that are toxic to rhizosphere fungi^26^. Unlike soil MBC, MBN concentration was not significantly different between N treatments and the control. This response of MBN to N enrichment may reflect a luxury N-uptake after addition of N. Contrarily, two other previous studies conducted in two bamboo plantations in vicinity of this study site showed that N addition significantly increased both MBC and MBN concentrations, and it was accompanied by an increase in root biomass^10, 31, 41^. We speculated that the response of root biomass to simulated N deposition may mainly determine the response of microbial biomass to N addition in this region.

In this study, we found a significant decrease in the soil MBC: MBN ratio. Baldos *et al*.^6^ demonstrated that N additions significantly reduced the soil MBC: MBN ratio at different elevations, and speculated that the decline in the soil MBC: MBN ratio corresponded to an increase in the ratio of bacteria to fungi. This is because the C: N ratio of bacteria is generally lower than that of fungi. Other previous studies also corroborated this hypothesis by demonstrating that the bacteria: fungi ratio was increased by N treatment^54^, or significantly and positively correlated with the level of N deposition^55^. This shift in the bacteria: fungi ratio suggests that N fertilization may have altered microbial community composition in our study as well. Further research is warranted to examine in depth the effects of N enrichment on microbial community structure at our study site.

A number of studies on N additions in forest ecosystems reported that the soil respiration rate decreased as a consequence of the decreases in root and soil microbial biomass^23–25, 56–58^. This parallel was detected in this study. In the present study, we found significant and positive correlations between soil CO_2_ emissions and both root biomass and soil MBC content (*r* = 0.595, *P* = 0.009; *r* = 0.630, *P* = 0.028, respectively). In other words, the decrease in root and soil microbial biomass was the likely instigator for the decrease in soil respiration under N addition.

In our study, experimental N additions had a non-significant influence on the contents of soil TOC and TN. Globally, N fertilization generally has an insignificant effect on soil C storage^59^. Theoretically, the addition of N has the potential to increase soil C content by boosting ecosystem net primary productivity^9, 11, 14^, by reducing the decomposition rate of soil organic matter (SOM)^60^, and by inhibiting soil respiration^24^. However, the decrease in underground C allocation detected in our study likely restricted any enrichment of soil C and N^15, 48^. Furthermore, because of the size and heterogeneous nature of the total soil C pool, a long experimental duration is necessary to observe the influence of N fertilization on soil C content^15^. For instance, Huang *et al*.^14^ conducted a 15-year-long field experiment in a second-rotation *Pinus radiata* plantation in New Zealand and observed that the surface soil C concentration of N-fertilized plots significantly increased after 10 years of treatment, but no significant change at the first 5 years. Therefore, it is possible that soil C and N contents at our study site may display increases over a long-term N addition experiment.

Our results indicate that artificial N additions had no significant influence on soil AP concentrations, which is consistent with a meta-analysis of Deng *et al*.^61^ who found that N additions had no significant effect on soil labile P across global sites. The insignificant increase in AP in N-treated plots may be explained by the AcPh activity. This is supported by the significantly positive correlation between soil AP content and AcPh activity, reported previously by Zheng *et al*.^62^ in two subtropical forests in southern China. In general, most N addition experiments reported increases in the activity of soil enzymes involved in P cycling^30, 31, 62^. This upregulation indicates that the transformation of organic P to inorganic P is accelerated by N additions, and thus may cause increases in soil AP. However, Tu *et al*.^31^ observed that soil AP concentration in a bamboo forest was significantly decreased by N fertilization, despite an increase in AcPh activity. According to Tu *et al*.^31^, the increased microbial biomass may promote the immobilization of inorganic P and thus result in a decrease in soil AP. However, in our study, microbial biomass was lowest in the HN treatment. It can be hypothesized that the microbial retention of inorganic P may decline as a consequence. This would lead to a small increase in soil AP concentration, which was indeed observed.

The influence of added N on soil enzyme activity can be attributed to the response of soil microbes, and soil properties such as SOM and pH^63^. Throughout the experimental period, N additions significantly reduced NR activity, but had no significant effects on the activities of urease, invertase, protease and AcPh. In a similar study in a subtropical forest, Wang *et al*.^30, 64^ observed that the addition of N in the form of NH_4_NO_3_ solution generally restricted NR activity, while other forms of N accelerated NR activity. Evidently, the formulation by which N is applied determines the effects on soil enzyme activity^64^. The decline in NR activity suggests that denitrification may have been restrained by NH_4_NO_3_ addition, given the role of NR as a crucial enzyme in denitrification. The negative correlation between NR activity and NO_3_^−^ concentration suggested that the loss of NO_3_^−^ via denitrification was limited by N addition. In our study, soil microbes may have contributed little to the changes in soil enzyme activity, since no significant correlations were observed. Instead, soil pH and nutrients played a more important role. Significantly, NR and AcPh activities were positively correlated to soil pH and TN concentration, and negatively correlated with TOC content. On the other hand, invertase, urease and protease activities were positively correlated to AK, AP and NO_3_^−^ concentration, respectively. These results indicate that N additions tend to increase N and P mineralization rates, but have little effect on C mineralization. Because of inherent variation in soil properties, microbial communities and functional diversity, vegetation type, N formulations, and experimental duration, it is difficult to draw a unified conclusion regarding the responses of soil enzyme activities to N addition.

In the present study, we found that litterfall mass was not correlated to N additions, a finding that is consistent with certain previous studies^20^, but not others^21, 65^. Nevertheless, it is worth noting that most previous studies detected that the N content and C: N ratio of litterfall respectively increased and decreased under N addition^20, 21, 65^.

## Conclusion

Artificial N deposition in the study area tended to increase nutrient availability. Conversely, N addition decreased root biomass and soil microbial biomass, thus slowing soil C emissions. The effects of N addition on soil pH and MBC/MBN indicated that soil acidification and altered microbial community size or even its composition were the primary results of increased N deposition. Microbial community composition as such was no investigated yet, and further research is needed.
